# Halicin: A New Approach to Antibacterial Therapy, a Promising Avenue for the Post-Antibiotic Era

**DOI:** 10.3390/antibiotics14070698

**Published:** 2025-07-11

**Authors:** Imane El Belghiti, Omayma Hammani, Fatima Moustaoui, Mohamed Aghrouch, Zohra Lemkhente, Fatima Boubrik, Ahmed Belmouden

**Affiliations:** 1 Laboratory of Cellular Biology and Molecular Genetics, Faculty of Sciences, Ibn Zohr University, Agadir 80000, Morocco; 2 Laboratory of Biological and Medical Analyses of the Hassan II Hospital, Agadir 80000, Morocco; 3 Laboratory of Medical-Surgical, Biomedicine and Infectiology Research, Faculty of Medicine and Pharmacy, Ibn Zohr University, Agadir 80000, Morocco

**Keywords:** halicin, antimicrobial resistance, artificial intelligence, repurposed drug, minimum inhibitory concentration, multidrug-resistant bacteria

## Abstract

**Background:** The global spread of antibiotic-resistant bacteria presents a major public health challenge and necessitates the development of innovative antimicrobial agents. Artificial intelligence (AI)-driven drug discovery has recently enabled the repurposing of existing compounds with novel therapeutic potential. Halicin, originally developed as an anti-diabetic molecule, has been identified through AI screening as a promising antibiotic candidate due to its broad-spectrum activity, including efficacy against multidrug-resistant pathogens. **Methods:** In this study, the antibacterial activity of halicin was evaluated against a range of clinically relevant multidrug-resistant bacterial strains. Bacterial isolates were first characterized using the agar disk diffusion method with a panel of 22 conventional antibiotics to confirm resistance profiles. The minimum inhibitory concentration (MIC) of halicin was then determined for selected isolates, including *Escherichia coli* ATCC^®^ 25922™ and *Staphylococcus aureus* ATCC^®^ 29213™, using broth microdilution according to Clinical and Laboratory Standards Institute (CLSI) guidelines. **Results:** Halicin demonstrated notable antibacterial activity, with MIC values of 16 μg/mL and 32 μg/mL against *E. coli* ATCC^®^ 25922™ and *S. aureus* ATCC^®^ 29213™, respectively. A dose-dependent inhibition of bacterial growth was observed for the majority of tested isolates, except for *Pseudomonas aeruginosa*, which exhibited intrinsic resistance. This lack of susceptibility is likely related to reduced outer membrane permeability, limiting the intracellular accumulation of halicin. **Conclusions:** Our findings support the potential of halicin as a novel antimicrobial agent for the treatment of infections caused by antibiotic-resistant bacteria. However, further investigations, including pharmacokinetic, pharmacodynamic, and toxicity studies, are essential to assess its clinical safety and therapeutic applicability.

## 1. Introduction

The global health threat posed by antimicrobial resistance (AMR) has reached alarming levels, with multidrug-resistant (MDR) bacteria increasingly rendering current treatment regimens ineffective. Among the most clinically relevant MDR organisms are the ESKAPE pathogens—*Enterococcus faecium*, *Staphylococcus aureus*, *Klebsiella pneumoniae*, *Acinetobacter baumannii*, *Pseudomonas aeruginosa*, and *Enterobacter* spp.—which are responsible for the majority of nosocomial infections and display a high degree of resistance to multiple antibiotic classes [1,2]. These pathogens have been prioritized by the World Health Organization for urgent research and the development of new antimicrobial therapies due to their capacity to “escape” the effects of conventional antibiotics [3].

Resistance to last-line antibiotics, particularly colistin and carbapenems, has become increasingly common among ESKAPE organisms. This trend poses a critical threat to public health as it severely limits the therapeutic arsenal available to clinicians, resulting in prolonged hospitalizations, higher treatment costs, and increased mortality rates [4,5]. Consequently, the discovery and development of new antimicrobial agents are of paramount importance. 

Historically, antibiotic discovery has relied heavily on screening natural products from soil-dwelling microorganisms, bioactive plant extracts, and synthetic compounds [6]. While these traditional approaches have led to the development of many clinically effective antibiotics, the pipeline of new antimicrobial agents has significantly slowed over the past decades. Additionally, these methods are time-consuming, costly, and yield diminishing returns as bacterial resistance mechanisms evolve faster than discovery pipelines can replenish [7].

Recent advances in artificial intelligence (AI) and machine learning (ML) have introduced a paradigm shift in drug discovery. These technologies are capable of processing vast chemical and biological datasets, recognizing complex patterns, and predicting bioactivity with unprecedented speed and accuracy. AI-based models can screen millions of molecules in silico, reducing both time and costs while accelerating hit identification [8]. A landmark example of this approach is the discovery of halicin, a compound originally developed as a c-Jun N-terminal kinase (JNK) inhibitor for diabetes and later repurposed as a potent antibiotic through deep learning algorithms developed at MIT [9].

**Figure 1:**
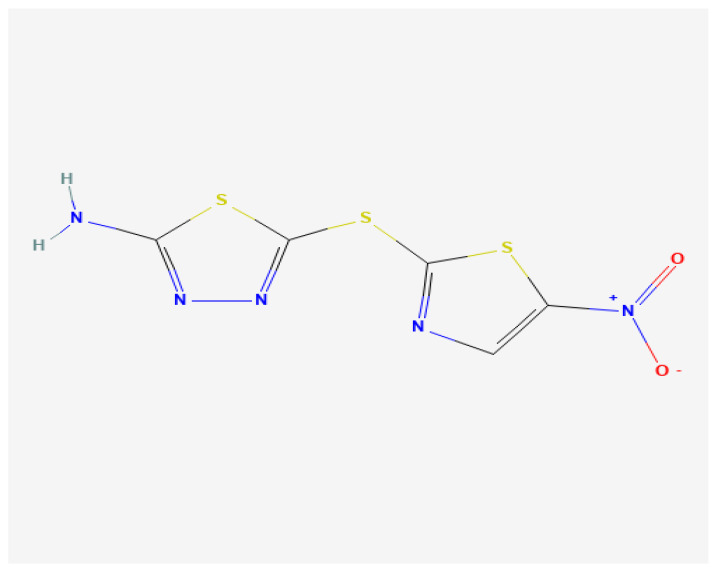
Halicin chemical structure. https://pubchem.ncbi.nlm.nih.gov/compound/Halicin#section=2D-Structure (accessed on 25 July 2024).

Halicin exhibits a broad-spectrum antibacterial activity distinct from classical antibiotics. Its mechanism involves disruption of the proton motive force across the bacterial membrane by interfering with iron homeostasis, thereby impairing ATP synthesis and essential transport processes [8]. The chemical structure of Halicin is presented in Figure 1 and was retrieved from the PubChem database.

Notably, halicin retains its efficacy against Gram-positive and Gram-negative MDR pathogens, including carbapenem-resistant *A. baumannii*, extended-spectrum β-lactamase (ESBL)-producing *E. coli*, and methicillin-resistant *S. aureus* (MRSA) [8]. Given these promising findings, halicin represents a compelling candidate for further antimicrobial development. However, detailed investigations into its activity against clinical MDR isolates remain limited, particularly in terms of its minimum inhibitory concentration (MIC) determinations for priority pathogens. Accurate MIC data are essential for defining effective therapeutic concentrations and evaluating clinical applicability.

In this study, we aimed to evaluate the in vitro antibacterial activity of halicin against a panel of MDR clinical isolates belonging to the ESKAPE group. We first screened the isolates using the disk diffusion method with 22 conventional antibiotics to characterize their resistance profiles, followed by the determination of halicin MIC values according to Clinical and Laboratory Standards Institute (CLSI) guidelines. Our goal was to contribute new data to the growing body of research on AI-discovered antimicrobials and explore halicin’s potential as a therapeutic alternative for drug-resistant infections.

## 2. Results

### 2.1. Agar Diffusion Test (Antibiogram) for MDR Strains Tested

Antibiotic susceptibility profiles of the clinical isolates were systematically assessed using the agar diffusion method (Kirby–Bauer disk diffusion), following the guidelines established by the Clinical and Laboratory Standards Institute (CLSI) [10]. The bacterial panel comprised multidrug-resistant (MDR) strains collected from diverse clinical sources, tested against a comprehensive antibiotic panel of 22 agents spanning multiple antibiotic classes, including β-lactams, aminoglycosides, fluoroquinolones, sulfonamides, nitrofurans, and carbapenems [11].

Our results revealed a notably high prevalence of resistance: approximately 88% of the isolates demonstrated resistance to at least one antibiotic within each tested class, confirming their multidrug-resistant phenotype. This extensive resistance across a broad spectrum of antibiotics highlights the critical challenge posed by these pathogens in clinical settings, consistent with global reports on the rise in MDR bacteria [12,13].

Conversely, a minority subset of isolates (~3%) exhibited partial susceptibility, primarily to nitrofurantoin, netilmicin, tobramycin, cotrimoxazole, levofloxacin, and imipenem. These antibiotics thus represent the few remaining therapeutic options showing some retained activity against select MDR strains within this cohort [14].

The predominance of multidrug resistance observed here aligns with trends reported for ESKAPE pathogens worldwide and underscores the urgent need for novel antimicrobial agents [15]. The limited efficacy of many frontline and last-resort antibiotics in this study further emphasizes the importance of exploring alternative therapeutic candidates, such as halicin, to address the escalating threat of MDR bacterial infections [8].

**Figure 2:**
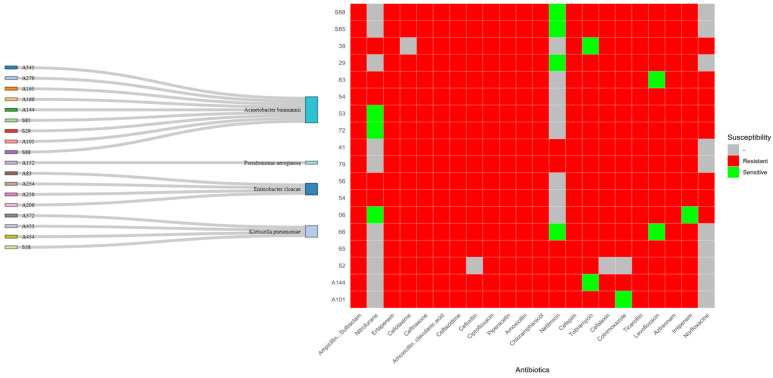
Antibiotic susceptibility test results for the 18 strains tested reveal a complex picture of antibiotic sensitivity and resistance. Red areas represent resistance to the tested antibiotics, highlighting the difficulties caused by multi-resistant bacteria in our sample. In contrast, green areas highlight the antibiotic sensitivity of the strains, providing potential treatment options for these infections. It is important to note that the gray areas represent antibiotics that were not tested for the indicated strains.

Figure 2 summarizes the distribution of susceptibility and resistance patterns for the clinical isolates tested.

### 2.2. Evaluation of the Antibacterial Activity of Halicin Against E. coli ATCC^®^25922™ and Staphylococcus aureus ATCC^®^ 29213™

**Figure 3:**
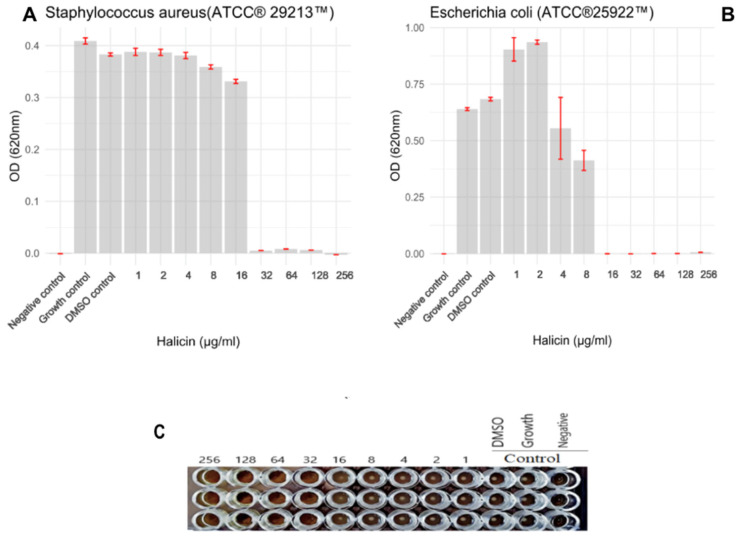
MIC determination using the broth microdilution method for (**A**)—*S. aureus* (ATCC 29213), (**B**)—for *E. coli* (ATCC 22925), the optical density was measured at 620 nm. Values are means of three replicates and error bars indicate the standard deviation. (**C**)—The photograph of a 96-well microdilution plate to determine the MIC for *S. aureus* (ATCC 29213), MIC = 32 µg/mL.

The antibacterial activity of halicin was evaluated in vitro against two standard reference strains—*Escherichia coli* ATCC^®^ 25922™ and *Staphylococcus aureus* ATCC^®^ 29213™—commonly used for quality control in antimicrobial susceptibility testing [16]. Our results demonstrated that halicin exhibited potent inhibitory activity, with minimum inhibitory concentrations (MICs) of 16 μg/mL for *E. coli* ATCC^®^ 25922™ and 32 μg/mL for *S. aureus* ATCC^®^ 29213™ (Figure 3). These findings highlight halicin’s broad-spectrum potential, effectively targeting both Gram-negative and Gram-positive organisms. Importantly, the MIC value obtained for *E. coli* ATCC^®^ 25922™ aligns with previous data reported in the literature. A 2021 study [17] similarly reported an MIC of 16 μg/mL for halicin against the same strain, supporting the reproducibility and consistency of halicin’s antibacterial effect. Although the MIC value of 16 µg/mL for *E. coli* ATCC^®^ 25922™ is higher than the clinical susceptibility thresholds defined for many conventional antibiotics, it remains encouraging in the context of Gram-negative bacteria, which are known for their restrictive outer membrane permeability. This result is consistent with previous findings [8] supporting the reproducibility of halicin’s antibacterial activity.

**Figure 4:**
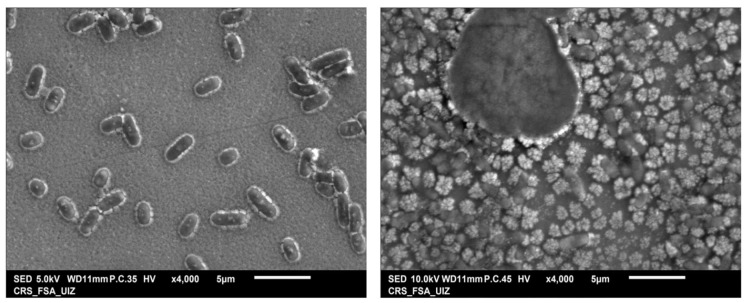
Result of SEM visualization of the effect of halicin on *E. coli* strain ATCC^®^25922™. (**left**): without halicin; (**right**): with halicin.

To further investigate the morphological impact of halicin on bacterial cells, scanning electron microscopy (SEM) was employed to visualize *E. coli* ATCC^®^ 25922™ following treatment with halicin. As illustrated in Figure 4, the SEM analysis revealed a marked reduction in the number of intact bacterial cells, along with visible membrane disruption and cell lysis, suggesting a loss of structural integrity. These observations are consistent with the proposed mechanism of action of halicin, which involves disruption of the electrochemical membrane potential through interference with iron homeostasis, ultimately leading to bacterial cell death.

Overall, these findings confirm the significant in vitro efficacy of halicin against well-characterized reference strains and underscore its potential as a promising candidate in the development of novel antimicrobial therapies targeting multidrug-resistant pathogens.

### 2.3. Evaluation of the Antibacterial Activity of Halicin Against Clinical Multidrug-Resistant Bacteria

**Figure 5:**
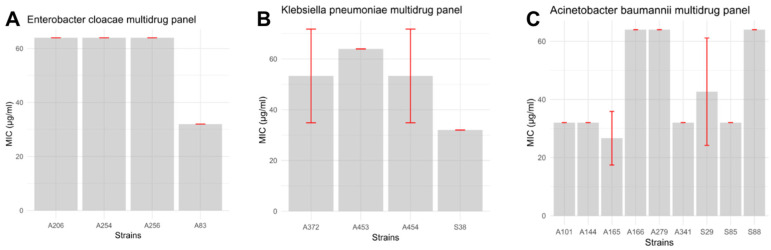
MIC determination using the broth microdilution method for clinical MDR strains: (**A**)—*E. cloacae*; (**B**)—*K. pneumoniae*; (**C**)—*A. baumannii*. Each strain is tested in three replicates.

The evaluation of halicin’s antibacterial activity against a panel of multidrug-resistant (MDR) clinical isolates revealed notable heterogeneity in the minimum inhibitory concentrations (MICs), even among isolates belonging to the same bacterial species (Figure 5). All tested strains were recovered from various clinical samples at the Hassan II Regional Hospital in Agadir and were internally coded for identification.

Among the *Acinetobacter baumannii* isolates, MIC values of 32 μg/mL were observed for strains A101, A144, S85, S29, A341, and A165, while higher MICs of 64 μg/mL were recorded for strains A272, S88, and A166. However, statistical analysis using the Kruskal–Wallis test did not reveal a significant difference in halicin susceptibility between these groups (*p* = 0.433), indicating relatively consistent activity within this species.

**Figure 6:**
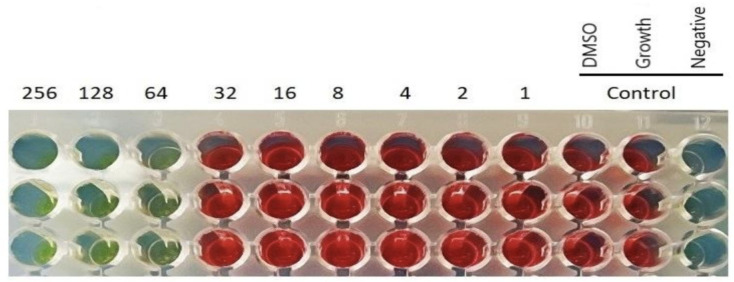
A photograph of a 96-well microdilution plate to determine the MIC for *E. cloacae* A254, MIC = 64 µg/mL. The visualization of the plates was achieved by adding 20 µL of triphenyl–tetrazolium chloride (TTC) solution (0.02 g mL^−1^) incubated at 37 °C for 2 h. The microbial growth was observed when the suspension color changed to red. Unchanged coloration indicates no bacterial growth.

Similarly, for *Enterobacter cloacae*, MICs of 64 μg/mL were noted for isolates A206, A256, and A254 (Figure 6), while isolate A83 exhibited a lower MIC of 32 μg/mL. Again, no statistically significant difference in halicin susceptibility was found among these strains (*p* = 0.392).

For *Klebsiella pneumoniae*, MIC values were predominantly 64 μg/mL for isolates A453, A454, and A372, whereas strain S38 exhibited a MIC of 32 μg/mL. No significant difference in MIC values was found in this group either (*p* = 0.392).

Overall, these findings suggest that while halicin exerts variable inhibitory effects depending on the isolate, its activity remains relatively consistent within species groups. Importantly, halicin demonstrated a clear, dose-dependent inhibitory effect against the majority of the clinical MDR isolates tested.

An exception to this trend was observed with *Pseudomonas aeruginosa* isolate A152, for which halicin exhibited no detectable antibacterial activity within the concentration range tested (1–256 μg/mL). This result suggests potential intrinsic or acquired resistance mechanisms in *P. aeruginosa* that warrant further investigation.

**Figure 7:**
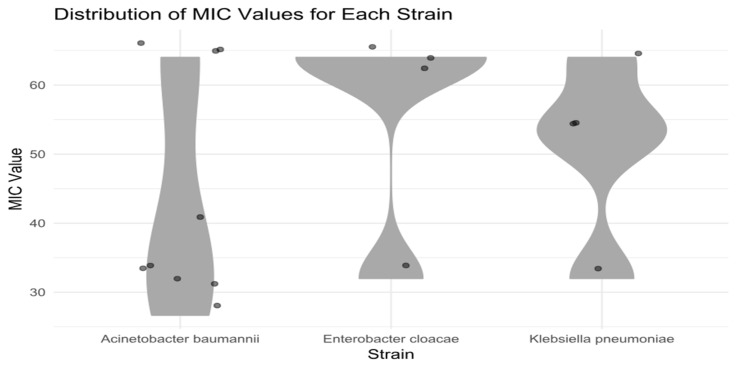
The distribution of MIC of clinical MDR strains: *E. cloacae*, *K. pneumoniae*, and *A. baumannii*.

The MIC distribution of halicin against all clinical MDR isolates tested—including *E. cloacae*, *K. pneumoniae*, and *A. baumannii*—is summarized in Figure 7. These results underscore the potential of halicin as an alternative therapeutic candidate, particularly against problematic MDR pathogens from the ESKAPE group, with the notable exception of *P. aeruginosa*.

## 3. Discussion

This study represents the first investigation in Morocco assessing the antibacterial activity of halicin against a panel of multidrug-resistant (MDR) Gram-negative bacteria isolated from clinical specimens. Our findings offer significant insights into the potential of halicin as a novel antibacterial agent, particularly in the context of growing antibiotic resistance in North Africa.

Halicin demonstrated measurable inhibitory activity against several Gram-negative pathogens, with MIC values ranging between 16 and 64 µg/mL. Specifically, halicin exhibited activity against *Escherichia coli* ATCC^®^ 25922™ and *Staphylococcus aureus* ATCC^®^ 29213™, with MICs of 16 µg/mL and 32 µg/mL, respectively. These values are consistent with previous work by Stokes et al., who initially described halicin’s potent activity against *E. coli* and other pathogens, reporting MICs of around 32 µg/mL for *E. coli* ATCC strains [8]. Other studies have since confirmed its broad-spectrum activity, including against carbapenem-resistant *K. pneumoniae* and *A. baumannii*, albeit with some variability in MIC values [18,19].

Interestingly, the variation in MICs observed among our clinical isolates—particularly for *A. baumannii*, *E. cloacae*, and *K. pneumoniae*—is not unexpected and may be attributed to several intrinsic and extrinsic factors. These include differences in outer membrane permeability, efflux pump expression, enzymatic inactivation, and the presence of resistance genes such as blaOXA, blaKPC, or blaNDM [20,21,22]. It is well established that the permeability of the bacterial envelope plays a crucial role in drug uptake, and limited permeability in MDR strains often correlates with higher MICs and reduced drug efficacy [23].

Furthermore, experimental conditions, including medium composition, inoculum size, incubation time, and solvent effects (such as DMSO stability), may contribute to discrepancies in MIC values across different studies [24,25]. The lack of significant statistical differences between some tested strains may reflect the limitations of phenotypic assays in capturing complex resistance mechanisms at the molecular level.

A particularly noteworthy result was the complete lack of activity of halicin against *Pseudomonas aeruginosa*. Even at the highest concentration tested (256 µg/mL), no inhibitory effect was observed. This aligns with findings from other research indicating high MIC values (>100 µg/mL) for *P. aeruginosa*, which are consistent with its intrinsic resistance mechanisms, including the expression of multidrug efflux pumps (e.g., MexAB-OprM), reduced porin channels (e.g., OprD), and a highly impermeable outer membrane [26,27,28]. These structural and regulatory barriers make *P. aeruginosa* particularly challenging to treat and underscore the need for combination therapies or targeted delivery strategies when using novel agents like halicin.

The mode of action of halicin appears to be distinct from that of traditional antibiotics. Stokes et al. proposed that halicin disrupts the electrochemical gradient across the bacterial membrane, leading to depolarization and subsequent cell death—a mechanism that is not readily circumvented by classical resistance pathways [8]. This unique activity may explain halicin’s broad-spectrum efficacy and its reported success against extensively drug-resistant (XDR) strains in murine models [29].

While our data are promising, several considerations must be addressed before clinical application. First, the pharmacokinetics and pharmacodynamics (PK/PD) of halicin in humans remain poorly understood. Preclinical studies have shown favorable results in mouse infection models, but extrapolation to human physiology requires careful dose optimization and safety evaluation [29,30]. Secondly, halicin’s stability in biological fluids, its interaction with host microbiota, and its potential cytotoxicity must be thoroughly evaluated [31].

Although the large-scale production of halicin may present certain technical challenges, it remains achievable. Initially identified from a library of repositioned compounds, halicin (formerly SU3327) is synthesized through a multi-step organic process involving the formation of a triazolo–thiadiazole core. The described synthetic route relies on relatively demanding reaction conditions, including elevated temperatures, the use of specific polar aprotic solvents, and metal catalysts. Reported yields for these steps in the article generally range between 40 and 60%, suggesting moderate performance compared to typical multi-step organic syntheses. These features indicate that further optimizations could improve the robustness and cost-effectiveness of the process for industrial-scale production. Moreover, the sequential isolation of intermediates and the use of costly or sensitive reagents are factors that may impact the overall efficiency of the synthesis [32].

Future developments in green chemistry, continuous-flow synthesis, and biosynthetic engineering could provide more sustainable, economically viable, and scalable strategies to facilitate large-scale halicin production.

In conclusion, our findings support the antimicrobial potential of halicin against several MDR Gram-negative pathogens, including *A. baumannii*, *E. cloacae* and *K. pneumoniae*, though its inefficacy against *P. aeruginosa* remains a significant limitation. Halicin holds promise as a next-generation antibiotic, but further investigations are essential to validate its safety, efficacy, and therapeutic potential in clinical settings.

## 4. Materials and Methods

**Preparation and Identification of Bacterial Isolates:** A total of 18 clinical multidrug-resistant (MDR) Gram-negative bacterial isolates were included in this study. The bacterial panel comprised *Enterobacter cloacae* (n = 4), *Acinetobacter baumannii* (n = 9), *Klebsiella pneumoniae* (n = 4), and *Pseudomonas aeruginosa* (n = 1). All isolates were previously collected from diverse clinical specimens at the Hassan II Regional Hospital in Agadir, Morocco. Initial bacterial identification was performed based on colony morphology on selective and differential media, followed by biochemical characterization using API 20E strips (bioMérieux, France), according to the manufacturer’s instructions. When necessary, supplementary identification techniques, including serotyping and antimicrobial susceptibility profiling, were conducted to confirm species and strain characteristics, in line with CLSI guidelines.

The isolates were stored at −80 °C in nutrient broth supplemented with 20% glycerol to preserve viability until further use. Reference strains *Escherichia coli* ATCC^®^ 25922™ and *Staphylococcus aureus* ATCC^®^ 29213™ were employed as quality controls in antimicrobial susceptibility assays.

**Preparation of Halicin Stock Solution:** Halicin (JNK Inhibitor XIII; CAS Number: 40045-50-9) was procured from Merck KGaA (Darmstadt, Germany). A stock solution of 20 mg/mL was prepared by dissolving 10 mg of Halicin in sterile dimethyl sulfoxide (DMSO; Sigma-Aldrich, St. Louis, MO, USA). Aliquots of the stock solution were stored at −20 °C in amber tubes to protect from light and minimize degradation. The chemical structure of Halicin was retrieved from the PubChem database and is presented in Figure 1.

**Determination of Minimum Inhibitory Concentrations (MIC):** The MICs of Halicin against the bacterial isolates were determined using the broth microdilution method, adapted from Wiegand et al [33]. with minor modifications. Overnight bacterial cultures were grown in nutrient broth at 37 °C and adjusted to a turbidity equivalent to 0.5 McFarland standard (optical density at 625 nm = 0.08–0.13), corresponding to approximately 1 × 10^8^ colony-forming units (CFU)/mL. These suspensions were subsequently diluted to 1 × 10^6^ CFU/mL in nutrient broth.

Serial two-fold dilutions of Halicin were prepared in sterile 96-well microtiter plates to final concentrations ranging from 256 µg/mL to 1 µg/mL. Each well was inoculated with 100 µL of diluted Halicin solution and 100 µL of bacterial inoculum, yielding a final bacterial concentration of ~5 × 10^5^ CFU/mL per well. Controls included wells with bacteria but no drug (growth control), wells containing medium with DMSO only (vehicle control), and medium alone (sterility control). All tests were performed in triplicate to ensure reproducibility.

Microplates were incubated at 37 °C for 18–20 h under constant agitation at 120 rpm to facilitate oxygenation and uniform exposure. Post incubation, bacterial growth was assessed spectrophotometrically by measuring absorbance at 620 nm using a Multiskan™ FC microplate reader (Thermo Fisher Scientific, Waltham, MA, USA). To further confirm viability, 20 µL of 2,3,5-triphenyl tetrazolium chloride (TTC, 0.02 g/mL) was added to each well, followed by incubation for 2 h at 37 °C. A reduction in TTC obtained through metabolically active bacteria produces a red color; the MIC was defined as the lowest concentration of Halicin that prevented visible growth (no color change) and showed an absorbance comparable to the sterility control.

**Scanning Electron Microscopy (SEM) Analysis:** To evaluate the morphological impact of Halicin on bacterial cells, treated bacterial suspensions were deposited on sterile glass slides and air-dried at room temperature. Samples were then mounted on aluminum stubs and coated with a thin gold layer using a sputter coater (JFC-1300, JEOL Ltd., Tokyo, Japan) to enhance conductivity. Morphological changes were examined using a scanning electron microscope (JSM-IT100, JEOL Ltd., Tokyo, Japan) at appropriate magnifications. Micrographs were captured digitally for comparative analysis between treated and untreated cells.

**Antimicrobial Susceptibility Testing Via Disk Diffusion:** Antibiotic susceptibility profiles of clinical isolates were determined by the Kirby–Bauer disk diffusion method on Mueller–Hinton agar plates (MH; BK048HA, BIOKAR, Pantin Cedex, France), following EUCAST guidelines. Bacterial inocula were standardized to 0.5 McFarland and evenly swabbed on agar plates. Antibiotic disks representing a panel of 22 agents across multiple classes were applied. Plates were incubated aerobically at 37 °C for 16–18 h, and inhibition zone diameters were measured and interpreted according to EUCAST breakpoints.

**Statistical Analysis:** All statistical analyses were conducted using RStudio software (version 4.3.3; R Foundation for Statistical Computing, Vienna, Austria). The Kruskal–Wallis non-parametric test was used to compare MIC distributions across bacterial species, with a significance threshold set at *p* < 0.05. Data visualization was performed using packages including ggplot2, reshape2, dplyr, purrr, and rstatix. Heatmaps and Sankey diagrams were generated with ggplot2 and networkD3, while bar plots with standard deviations and violin plots were constructed using ggplot2.

## 5. Conclusions

This study could thus provide a reference point for evaluating the antibacterial efficacy of Halicin and for determining its pharmacological and toxicological profiles. Given the ability of bacteria to develop resistance to antibiotics, it is also essential to closely monitor the emergence of resistance to Halicin. It is essential to set up bacterial resistance monitoring programs to track changes in the susceptibility of bacterial strains to halicin. Such programs would enable the early detection of any changes in bacterial susceptibility to this innovative antibiotic and allow for appropriate measures to be taken to prevent the emergence of resistance. To date, no development of halicin resistance has been observed, mainly due to its limited and controlled use. However, without continuous and rigorous monitoring, there is a risk that resistance could emerge with time and increased use. The implementation of these programs is therefore crucial to ensure the long-term efficacy of halicin and to contribute to the global fight against multidrug-resistant (MDR) bacteria.

## Data Availability

The authors confirm that the data supporting the findings of this study are available within the article.
